# Similar Replicative Fitness Is Shared by the Subtype B and Unique BF Recombinant HIV-1 Isolates that Dominate the Epidemic in Argentina

**DOI:** 10.1371/journal.pone.0092084

**Published:** 2014-04-11

**Authors:** Andrea E. Rubio, Awet Abraha, Crystal A. Carpenter, Ryan M. Troyer, Ángel L. Reyes-Rodríguez, Horacio Salomon, Eric J. Arts, Denis M. Tebit

**Affiliations:** 1 Centro Nacional de Referencia para el SIDA, Departamento de Microbiología, Facultad de Medicina Universidad de Buenos Aires, Buenos Aires, Argentina; 2 Division of Infectious Diseases and HIV Medicine, Case Western Reserve University, Cleveland, Ohio, United States of America; Institut Pasteur, France

## Abstract

The HIV-1 epidemic in South America is dominated by pure subtypes (mostly B and C) and more than 7 BF and BC recombinant forms. In Argentina, circulating recombinant forms (CRFs) comprised of subtypes B and F make up more than 50% of HIV infections. For this study, 28 HIV-1 primary isolates were obtained from patients in Buenos Aires, Argentina and initially classified into subtype B (n = 9, 32.1%), C (n = 1, 3.6%), and CRFs (n = 18, 64.3%) using partial *pol* and *vpu-env* sequences, which proved to be inconsistent and inaccurate for these phylogenetic analyses. Near full length genome sequences of these primary HIV-1 isolates revealed that nearly all intersubtype BF recombination sites were unique and countered previous “CRF” B/F classifications. The majority of these Argentinean HIV-1 isolates were CCR5-using but 4 had a dual/mixed tropism as predicted by both phenotypic and genotypic assays. Comparison of the replicative fitness of these BF primary HIV-1 isolates to circulating B, F, and C HIV-1 using pairwise competitions in peripheral blood mononuclear cells (PBMCs) indicated a similarity in fitness of these BF recombinants to subtypes B and F HIV-1 (of the same co-receptor usage) whereas subtype C HIV-1 was significantly less fit than all as previously reported. These results suggest that the multitude of BF HIV-1 strains present within the Argentinean population do not appear to have gained replicative fitness following recent B and F recombination events.

## Introduction

Human immunodeficiency virus type 1 (HIV-1) evolved from the simian immunodeficiency virus (SIVcpz) predominant in the subspecies of chimpanzee, *Pan troglodytes troglodytes (Ptt*) [1]–[4]. The transmission events from apes to humans occurred on at least four separate occasions which is represented by phylogenetic distinct groups termed M (major), O (outlier), N (non-M, non-O) and P. HIV-1 group M is responsible for the global pandemic shown clearly by its divergence into nine “pure” clades or subtypes (A-D, F-H, J and K), 63 circulating recombinant forms (CRF) (www.hiv.lanl.gov) and a myriad of unique recombinant forms (URF). Globally, CRFs account for about 10–20% of all new HIV-1 infections while URFs are responsible for over 30% of infections in regions where two or more subtypes co-circulate [2], [3]. Intersubtype recombinants are clearly contributing to HIV-1 evolution and may ultimately result in complete disappearance of the “pure” HIV-1 subtypes [5], which in fact originated from earlier SIV/HIV recombination events [6].

**Figure 1:**
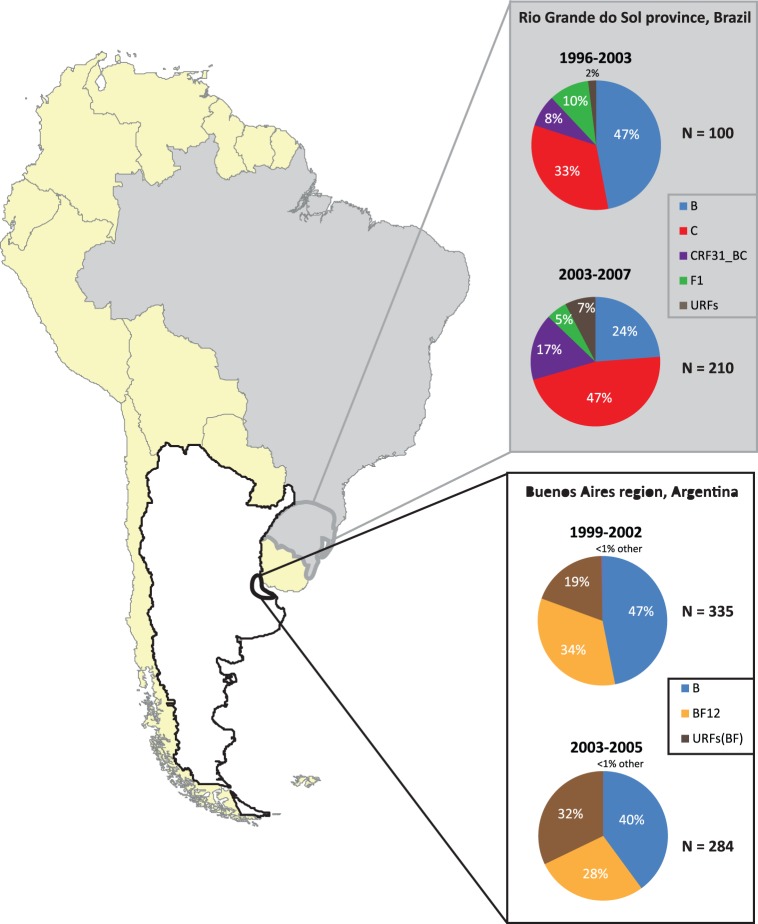
Changes in the prevalence of HIV-1 subtypes and recombinant forms during the past ten years in Buenos Aires, Argentina (white) and Rio Grande do Sol, Brazil (gray). Rio do Sol is located in the most southern part of Brazil while Buenos Aires is in north eastern Argentina. Both regions are located close to each other and share a common characteristic in that they harbor dominant and evolving recombinant forms. The prevalence numbers shown were obtained by combining the subtype distribution from published reports originating in these 2 regions. Only samples with a known sampling date were included in the analyses.

The prevalence of HIV-1 subtypes and CRFs in the human population varies by geographical region and can be influenced by various socio-cultural factors as well as virus-host genetics (founder effects and host restrictive factors) [7]. Although subtype B is dominant in developed countries and is the most widely studied clade, non-subtype B HIV-1 are responsible for >90% of HIV infections [3], [4] Subtype C dominates in prevalence (>50% of infections worldwide) due in part to epidemics in India, China, and southern African countries (South Africa, Zimbabwe, Botswana, Zambia, Namibia, Malawi) [3], [4]. In South America, the HIV-1 epidemic has been well characterized with subtype B HIV-1 found in the northern Pacific and Caribbean coastal countries (Venezuela, Columbia, Ecuador, Peru) whereas in the Southern cone (i.e. Argentina, Chile, Paraguay, and Uruguay) the epidemic is more diverse with a higher HIV-1 prevalence. In southern Brazil and Argentina [8], [9] subtype B, C, and F as well as B/C and B/F recombinants are the most prevalent (Figure 1).

Intersubtype BF recombinants were first reported as a CRF (CRF12_BF) in Argentina and Uruguay in 2001 [10], followed by other novel BF CRFs in Brazil, Uruguay and Chile (i.e. CRF17_BF, [10]; CRF28_BF, CRF29_BF [11]; CRF38_BF [12]; CRF39_BF, CRF40_BF [13], CRF44_BF; CRF46_BF [14]. CRF12_BF, CRF38_BF, and related BF recombinants appear to have originated in the late 1970s and early 1980s, dates which coincide with the beginning of the local epidemic [15], [16]. In the HIV-infected population of Brazil (with the exception of southern Brazil; Figure 1), subtype B (71%) dominates over a lower prevalence of intersubtype BF recombinants (12%), subtype C (10%), subtype F (5%) and other subtypes/intersubtypes (2%) [17]–[19]. In contrast, intersubtype BF recombinant (56.7%) are found at high prevalence in HIV-infected Argentineans with subtype B (34.5%), F and other subtypes/intersubtypes (<5%) observed at low frequency [20]–[23] (Figure 1). About 50% of these Argentinean intersubtype BF-like viruses are related to, but distinct from CRF12_BF [10], [20], [22]. Notably, most BF recombinants appear unique, but share a number of breakpoints with CRF12_BF [22]. Thus, previous or continuous super-infections involving CRF12_BF and/or other B, F, or BF recombinant viruses results in circulation of multiple BF recombinants which may be better defined as unique recombinant forms (URFs) rather than CRFs. This subtype/recombinant diversity and dynamics is quite unique to the Argentinean epidemic. In this study, the full genome sequences revealed that all of the 18 BF recombinant HIV-1 isolates from Buenos Aires, Argentina were URFs and not CRFs.

Based on the dominance of BFs in the Argentinean epidemic, Sanabani et al. (2006) [24] have proposed that these recombinants may have higher replicative fitness than “pure” subtypes B and F. However, aside from the fitness studies of CRF02_AG versus subtype A or G, there are no other comparisons of replicative fitness between recombinant HIV-1 and their parental isolates. Two studies have examined potential phenotypic differences among BF viruses [25], [26] and suggested that HIV-1 BF strains might have a higher replicative fitness over subtypes B and F HIV-1 isolates. In an effort to understand the high prevalence of intersubtype BF recombinants in Argentina, we compared *ex vivo* ‘pathogenic’ or replicative fitness of primary HIV-1 isolates of subtype B, F, C, and intersubtype BF recombinants derived from chronically infected patients. Fitness of these primary HIV-1 isolates was determined by direct head-to-head virus competitions in human peripheral blood cells (PBMCs) of HIV-negative donors. We discovered that the BF recombinants were of equal or lower replicative fitness compared with Argentinean B and reference F HIV-1 isolates.

## Materials and Methods

### Ethics Statement

Confidential one-on-one interviews were conducted onsite by health care workers. During these encounters, the study was explained and subjects were invited to participate. Only those subjects who were willing to participate were provided with a written informed consent, enrolled, and sampled. All procedures were approved by the Independent Research Ethics Committee, School of Medicine, University of Buenos Aires (CIEI-FM-UBA), Office for Human Research Protection (OHRP) reference numbers IORG#0004063 and IRB#00004817.

### Patients, Sample Collection, Viral Load Determination and CD4 Lymphocytes Count

Patients were sampled from September 2002 to July 2004 from two medical facilities in Buenos Aires: namely the *Hospital de Agudos José María Ramos Mejía*, (n = 15), a voluntary counseling and testing center where routine HIV testing is performed and the “National Reference Center for AIDS, School of Medicine, University of Buenos Aires” (n = 13) where patients are monitored routinely for viral load and CD4 cell count as standard-of-care. Subjects attending these centers were from the city of Buenos Aires and nearest suburbs. Clinical records were reviewed to confirm that these patients were antiretroviral naïve.

Whole blood samples were collected and an aliquot was used for CD4 determination. Briefly, absolute count of CD4^+^ T-lymphocytes (cells/ul) from peripheral whole blood was determined in each HIV-1-infected patient by flow cytometry using a Coulter XL (Beckman) instrument (**Table 1**). Remaining blood was separated into plasma and buffy coat. Viral load in plasma was assessed by the branched DNA technology [VERSANT^®^ HIV-1 RNA 3.0 Assay (b-DNA)] with a detection range of 50–500,000 HIV-1 RNA copies/ml (**Table 1**).

**Table 1:** Characteristics of Argentinean HIV-1 primary isolates and reference strains.

Isolate name	Subtype *Pol*	Subtype *Env*	Age (years)	Viral load (copies/mL)	CD4 (cells/μL)	Coreceptor usage	Phenotype (NSI/SI/DM)	Country of Origin
B271	B	B	59	175,113	121	R5	NSI	Argentina
B524	B	B	36	409,639	87	R5	NSI	Argentina
B542	B	B	34	123,704	nd	R5	NSI	Argentina
B552	B	B	40	nd	171	X4	SI	Argentina
B563	B	B	34	46,719	nd	R5	NSI	Argentina
B735	B	B	27	63,237	238	R5	NSI	Argentina
B775	B	B	37	2,802	nd	X4	SI	Argentina
B872	B	B	55	>500,000	nd	R5	NSI	Argentina
B958	B	B	27	28,838	318	R5	NSI	Argentina
B F027	BF	BF	36	127,093	150	R5×4	DM	Argentina
BF116	BF	BF	38	112,521	236	R5	NSI	Argentina
BF118	BF	BF	42	344,685	255	R5	NSI	Argentina
BF119	BF	BF	36	26,921	nd	R5	NSI	Argentina
BF134	BF	BF	25	18,384	nd	R5×4	DM	Argentina
BF303	BF	BF	42	199,019	280	R5	NSI	Argentina
BF329	B	BF	48	239,552	217	R5	NSI	Argentina
BF333	BF	BF	51	339,539	78	R5	NSI	Argentina
BF456	BF	BF	34	7,485	nd	R5	NSI	Argentina
BF461	BF	BF	36	68,750	nd	R5	NSI	Argentina
BF549	BF	F	34	68,421	nd	R5	NSI	Argentina
BF555	BF	BF	36	176,805	nd	R5×4	DM	Argentina
BF559	B	BF	38	>500,000	nd	X4	SI	Argentina
BF640	BF	BF	29	130,651	228	R5×4	DM	Argentina
BF714	B	BF	34	3,825	nd	R5	NSI	Argentina
BF819	BF	BF	46	27,317	nd	X4	SI	Argentina
BF955	BF	BF	30	>500,000	47	R5	NSI	Argentina
BF992	BF	BF	32	451,371	519	R5	NSI	Argentina
C674	C	C	51	nd	nd	R5	NSI	Argentina
97ZA003	C	C	na	nd	nd	R5	NSI	South Africa
96USNG58	C	C	na	na	na	R5	NSI	Nigeria
93MW959	C	C	na	na	na	R5	NSI	Malawi
VI850	F1	F1	na	na	na	R5	NSI	DRC
CA20	F2	F2	na	na	na	R5	NSI	Cameroon
93BR020_1	F1	F1	na	na	na	X4	SI	Brazil

DRC: Democratic DRC: Republic of Congo, na: not applicable; nd: not done.

### Virus Isolation, Co-receptor Usage and Titration

PBMCs isolated from HIV infected patients were co-cultivated with uninfected PBMCs from a healthy donor, as described previously [27]. Briefly, patient and uninfected cells were separately stimulated with 2 μg/ml phytohemagglutinin (PHA; Gibco, BRL) in RMPI medium for 2 to 3 days, and further maintained in complete RPMI medium containing 10% fetal bovine serum, 1 ng of recombinant interleukin (IL-2 invitrogen), 100 U/ml penicillin, and 100 μg/ml streptomycin (Cellgro)]. Subsequently, 1.5×10^6^ stimulated cells from uninfected donors were co-cultured, in 24-well tissue culture plates, with 1.5×10^6^ PBMC from HIV-1-infected patients. On day 4, 7, 11, 14 and 18, supernatant was harvested from each well to assess radioactive reverse transcriptase (RT) activity. On day 7 and 14, 1.5×10^6^ PHA-stimulated PBMC from the HIV-negative donor were thawed from frozen aliquots (derived from the same donor and blood draw) and added to each well/co-cultivation. Cell-free supernatants were harvested at peak viremia, as measured by a RT assay, centrifuged and stored at −80°C until subsequently needed. Twenty eight primary HIV-1 isolates from Argentina were obtained from this process. Two subtype F reference strains CA20 and VI850 were kindly provided by Dr. Guido Vanham (Institute of Tropical Medicine in Antwerp, Belgium). The other subtype reference strains (C-97ZA003, C-96USNG58, C-93MW959, F1-093BR020_1) were obtained from the National Institutes of Health AIDS Research and Reference Reagent Program (Table 1).

To determine co-receptor usage, U87 cell lines (human glioma cell line) expressing CD4 and either CCR5 or CXCR4 chemokine receptor (obtained from D. Littman and the AIDS Research and Reagent Program) were maintained in Dulbecco Modified Eagle Medium (DMEM; Cellgro) supplemented with 15% FBS, 100 U/ml penicillin, 100 mg of streptomycin, and 300 μg/ml geneticin (G418; Gibco-BRL) and 1 μg/ml puromycin (Life Technologies, Inc.) to retain receptor and co-receptor expression, respectively.{Deng, 1996 5706/id} Co-receptor usage was then assessed by infecting 2.5×10^3^ U87.CD4 cells expressing either CCR5 or CXCR4, in parallel, with each viral stock, in 48-well plates, as described previously [28]. Virus production was determined by assaying RT activity in the cell-free supernatants at days 3, 5, 7 and 9. Finally, CXCR4-usage was confirmed by infection of the HTLV-1-transformed MT2 cell line as described [29] (**Tables 1** **and** **2**).

**Table 2:** Analysis of viral tropism.

Virus strain	U87CD4 cells			Glycosite 301												11	GPGR Tip of V3 Loop Gly 312 to Arg 315															25												Net charge	11/25 rule	PSSM	g2p
	CCR5	CXCR4	MT2	295	296	297	298	299	300	301	302		303	304	305	306	307	308	309	310	311	312	313	314	315	316	317	318	319	320	321	322		325	326	327	328	329	330	331	332	333	334				
HXB2				N	C	T	R	P	N	N	N	−	T	R	K	R	I	R	I	Q	R	*G*	*P*	*G*	*R*	A	F	V	T	I	G	K	−	I	G	N	M	R	Q	A	H	C	N				
***B271***	+	−	−	.	.	.	.	.	N/G/S/D	.	.	−	.	.	.	**S/G**	.	H/P	−	−	I	**.**	**.**	**.**	***R/S***	.	.	Y	A	T	.	**D**	I/V	I/V	.	D	I/P/T/L	.	.	.	.	.	.	3.8	R5	R5	R5
***B524***	+	−	−	.	.	.	.	.	.	.	.	−	.	.	R	**S**	.	P	−	−	M	**.**	**.**	**.**	**.**	.	M/L	F	I	T/A	.	**D**	I	.	.	D	I	.	.	.	H/Y	.	.	3.5	R5	R5	R5
***B542***	+	−	−	.	.	S	.	.	.	.	.	−	.	.	K/R	**S**	.	P	−	−	M	**.**	**.**	**.**	**.**	.	.	Y	A	T	.	**D**	I	.	.	D	I	.	Q/R	.	Y	.	T	3.5	R5	R5	R5
***B563***	+	−	−	.	.	.	.	.	S	.	.	−	.	S	.	**S**	.	H	−	−	M	***A***	***F***	**.**	**.**	.	.	Y	A	T	E	**R**	I	.	.	D	I	.	K	.	.	.	S	6.0	X4	R5	R5−X4
***B735***	+	−	−	.	.	.	.	.	.	.	.	−	.	.	R	**S**	.	P	−	−	M/I	**.**	**.**	**.**	***K***	.	.	Y	A	T	.	**D**	V	.	.	D	I	.	.	.	Y/H	.	T	3.5	R5	R5	R5
***B872***	+	−	−	K	.	I	.	.	.	.	.	−	.	.	.	**S**	.	H	−	−	I	***A***	**.**	**.**	**.**	.	.	Y	A	T	.	**D**	I	.	.	D	I	.	.	.	.	.	I	5.0	R5	R5	R5
***B958***	+	−	−	.	.	.	.	.	.	.	.	−	.	.	R	**S**	.	H	−	−	L	**.**	**.**	**.**	***G***	T	.	Y	A	A	.	**E**	I	.	.	D	I	.	K	.	.	.	T	5.0	R5	R5	R5
**BF116**	+	−	−	T/N	.	.	.	.	.	.	.	−	.	.	K/T	**S**	.	H	−	−	L	**.**	***P/L***	**.**	**.**	.	.	Y	A	T	.	**D**	I	.	.	D	I	.	K	.	.	.	.	5.5	R5	R5	R5
**BF118**	+	−	−	.	.	.	.	N	.	.	.	−	.	.	.	**S**	.	P/S	−	−	I	**.**	**.**	**.**	**.**	.	.	Y	A	T	.	**D**	I	.	.	D	I	.	K	.	.	.	.	5.0	R5	R5	R5
**BF119**	+	−	−	.	.	.	.	.	.	.	.	−	.	.	.	**S**	.	P	−	−	M	**.**	**.**	**.**	***Q***	.	L	Y	V	T	.	**D**	I	.	.	.	I	.	K	.	.	.	.	5.0	R5	R5	R5
**BF303**	+	−	−	.	.	.	.	.	.	.	.	−	.	.	K/T	**S**	.	Q	−	−	L	**.**	**.**	**.**	**.**	.	.	Y	A	T	.	**D**	I	.	.	D	I	.	K	.	Y	.	N/T	3.5	R5	R5	R5
**BF329**	+	−	−	.	.	.	.	.	.	.	.	−	.	.	.	**S**	.	Q	−	−	I	**.**	**.**	**.**	**.**	.	.	Y	A	T	.	**D**	I	.	.	D	I	.	R	.	.	.	.	5.0	R5	R5	R5
**BF333**	+	−	−	.	.	.	.	.	.	.	.	−	.	.	.	**S**	.	H	−	−	L	**.**	**.**	**.**	***Q***	.	.	Y	A	T	.	**D/G/E**	I	.	.	.	I	.	K	.	H/Y	.	.	5.8	R5	R5	R5
**BF456**	+	−	−	N	.	.	.	.	N	.	.	−	.	.	E	**S**	.	H	−	−	I	**.**	**.**	**.**	**.**	.	.	Y	A	T	.	**D**	I	.	.	D	I	.	R	.	.	.	.	4.0	R5	R5	R5
**BF461**	+	−	−	S	.	I	.	.	.	.	.	−	.	.	.	**S**	.	H	−	−	L	**.**	**.**	**.**	***Q***	.	.	Y	A	T	.	**D**	I	.	.	D	I	.	.	.	.	.	K	4.0	R5	R5	R5
**BF549**	+	−	−	.	.	.	.	.	.	.	.	−	.	.	.	**S**	.	Q	−	−	M	**.**	**.**	**.**	**.**	.	.	Y	A	T	.	**D**	I	V	.	D	I	.	K	.	.	.	.	5.0	R5	R5	R5
**BF714**	+	−	−	.	.	S	.	.	.	.	.	−	.	.	.	**S**	.	Q	−	−	I	**.**	**.**	**.**	**.**	.	.	Y	A	T	.	**E**	I	.	.	D	I	.	K	.	.	.	.	5.0	R5	R5	R5
**BF955**	+	−	−	T	.	.	.	.	.	.	.	−	.	.	.	**S**	.	Q	−	−	L	**.**	**.**	**.**	***R/K***	.	I	Y	A	T	.	**D**	I	.	.	D	I	.	K	.	.	.	.	5.0	R5	R5	R5
**BF992**	+	−	−	.	.	.	.	.	.	.	.	−	.	K	.	**S**	.	P	−	−	I	***R***	**.**	**.**	***Q***	.	L	Y	V	T	E	**D**	I	.	R	.	I	.	K	.	.	.	.	6.0	R5	X4	R5
C674	+	−	−	.	.	.	.	.	.	.	.	−	.	.	.	**S**	.	.	−	−	I	**.**	**.**	**.**	***Q***	T	.	Y	A	T	.	**D**	I	.	.	D	I	.	.	.	.	.	.	4.0	R5	R5	R5
***B552***	−	+	+	.	.	.	.	.	Y	.	T	I	K	.	R	**I**	M	H	−	−	I	**.**	**.**	**.**	**.**	.	.	Y	A	T	−	**T**	G	P	.	D	I	.	R	.	Y	.	T	7.0	R5	X4	X4
***B775***	−	+	+	.	.	.	.	.	G	.	K	−	.	Q	R	**R**	L	S	−	−	I	**.**	**.**	**.**	**.**	.	.	.	A	T	R	**A**	I	T	.	D	P	.	R	.	.	.	T	8.0	X4	X4	X4
**BF559**	−	+	+	.	.	.	.	.	.	.	.	−	.	.	.	**G**	.	.	−	−	V	**.**	**.**	**.**	**.**	T	I	Y	A	T	E	**K**	I	.	.	D	I	.	K	.	.	.	.	7.0	X4	X4	X4
**BF819**	−	+	+	.	.	.	.	.	.	.	.	−	.	.	.	**G**	.	Q	−	−	L	**.**	**.**	**.**	***K***	V	.	Y	A	T	.	**E**	I	.	.	D	I	.	K	.	.	.	.	5.0	R5	R5	R5
**BF027o**	+	+	+	.	.	.	.	.	.	.	.	−	.	.	.	**S/G**	.	Q/H	−	−	L	**.**	**.**	**.**	***R/Q***	A/V	.	Y	A	T	.	**D**	I	.	.	N/D	I	.	K	.	.	.	.	5.5	R5	R5	R5
**BF134o**	+	+	+	.	.	.	.	.	.	.	.	−	T/I	.	.	**S**	.	H	−	−	M	**.**	***G***	**.**	**.**	.	F/L	Y	.	N	.	**Q**	I	.	.	.	I	.	.	.	H	.	.	7.0	R5	R5−X4	R5
**BF555o**	+	+	+	N/I	.	A	.	.	.	.	.	-	.	.	.	**S**	L	.	−	−	I	**.**	**.**	**.**	**.**	A/T	I	Y	A	T	.	**R**	I	V	.	D	I	.	.	.	.	.	.	7.0	X4	X4	X4
**BF640o**	+	+	+	.	.	.	.	.	.	.	.	−	.	.	.	**G**	.	H	−	−	I	***G/R***	**.**	**.**	**.**	.	.	Y	A	T	.	**S**	I	.	.	D	I	.	K	.	.	.	.	7.5	R5	R5	R5

Tropism of each viral isolate was assessed by infecting U87.CD4 cells expressing either CCR5 or CXCR4, in parallel, with each viral stock and by detection of syncytia on MT2 cell line. To further confirm viral tropism, V3-loop sequence of the gp120 protein of each sample was also evaluated in terms of net amino acid charge (net charge), the presence of positively charged amino acids at codons 11 and/or 25 (bold) of V3-loop (bold italics; HXB2 gp160 positions 306 and 322, respectively) (11/25 rule), and the two major bioinformatics algorithms Position-Specific Scoring Matrix (PSSM) and geno2pheno [coreceptor] (g2p). The 20 CCR5, 4 CXCR4-tropic and 4 dual/mixed-tropic isolates (o) are shown on the table. Sample identifications are represented as follows: subtype B (*bold italic*), C (normal) and BF (bold). All sequences were obtained from the original PBMC of patients.

For net charge, 11/25 rule, PSSM and g2p all possible amino acidic combinations according to amino acid change due to base ambiguities were evaluated. R5-X4: sample B563’s FPR value fell between the two cutoffs, evidencing the presence of X4 variants as well. R5-X4: for sample BF134o three out of four possible amino acidic combinations according to amino acid change due to base ambiguities were R5.

The tissue culture infectious dose for 50% infectivity (TCID_50_) values were determined by serially diluting each stock of HIV-1 isolate in triplicate and infecting 10^5^ stimulated PBMCs in a 96-well flat-bottom plate. Infectivity in each well was tested by the radiolabeled RT assay [28], and the TCID_50_ assay end point was determined on day 10. TCID_50_ values was calculated as infectious units per milliliter (IU/ml) using the Reed and Muench method [30].

### DNA Extraction, PCR, Sequencing and Phylogentic Analysis

DNA was extracted from PBMC of each HIV-1-infected patient with the QIAamp DNA Blood Mini Kit (Qiagen). Nested PCR was performed on extracted DNA to obtain several non-contiguous fragments spanning various regions of the HIV-1 genome or the full length genome of isolates. Primers used for these amplifications are available upon request. HIV-1 PCR products were purified using the Qiaquick DNA purification kit (Qiagen) and then sequenced in both directions using an ABI PRISM 3100 Genetic Analyzer (Applied Biosystems). Chromatograms were manually edited for alignment by using Sequencher 4.10.1 Software (Gene Codes) and Vector NTI suite.

Nucleotide sequences were aligned using Clustal X Multiple Alignment neighbor joining protocol version 7.0.5.3. Alignments included a representative set of known HIV-1 subtype reference sequences from the Los Alamos HIV Sequence Database. Neighbor-joining (NJ) trees were constructed under the Kimura 2-parameter model with the MEGA3 program. The codon alignment was performed using Gene Cutter tool available at the Los Alamos National Laboratory website (www.hiv.lanl.gov/content/sequence/GENE_CUTTER/cutter.html). Each HIV sequence was analyzed for recombination patterns using Simplot version 3.5.1 [31] with a bootscan sliding window of 300 nucleotides in 20 nucleotide steps (100 bootstrap replicates 50% consensus) [32]. Only sequences with bootstrap values above 75% throughout the scanned sequence and in which a fragment of >200 bp was shown to belong to a discordant subtype were considered recombinant forms. The Genotyping tool at the National Center for Biotechnology Information [33] and the jumping profile Hidden Markov Model (jpHMM) [34] were used to confirm recombination breakpoints. *pol* gene sequence from each sample (2253–3749, HXB2 numbering) was analyzed for drug resistance mutations, using the Stanford University calibrated population resistance tool (http://cpr.stanford.edu/cpr/), which provides a suitable approach for general batch-analysis of HIV-1 *pol* gene sequences (Table S1).

### Sequence Data

Sequences generated from this study have been submitted to the GenBank under the accession numbers KJ569150–KJ569178.

### Genotypic Determination of Viral Tropism

In order to predict genotypic viral tropism and confirm phenotypic co-receptor usage, we analyzed V3-loop sequences from each sample (HXB2 gp160 amino acids 296–334) using the Position-Specific Scoring Matrix (PSSM) (http://indra.mullins.microbiol.washington.edu/webpssm/) and geno2pheno [co-receptor] (g2p) (http://coreceptor.bioinf.mpi-inf.mpg.de/index.php) as well as detecting the presence of positively charge amino acids at codons 11 and/or 25 of the V3-loop (HXB2 gp160 positions 306 and 322, respectively). PSSM analysis was performed using subtype B X4R5 matrix whereas for g2p, significance levels were set to the optimized cutoffs based on clinical analyses from MOTIVATE (2% and 5.75% false positive rates) [35].

### *Ex vivo* Growth Competition Assays

The *ex vivo* pathogenic fitness of each virus was determined by performing duplicate pair wise dual infection/competitions experiments as described previously [36]. Briefly, Argentinean HIV-1 primary isolates were competed against each other as well as subtype reference strains (Table 1). Virus was added alone or in pairs to 2×10^5^ PHA/IL-2 PBMC from HIV-1-seronegative healthy donors at an equal multiplicity of infection (MOI) of 0.0005 (IU/cell) [36], [37]. All PBMCs for these fitness studies and for TCID_50_ determination were obtained from one donor and one blood draw of 500 ml. Assays were carried out in duplicates in 48-well cell culture plates. Full pair wise competitions were performed involving R5 vs R5-using isolates or X4 vs X4-using isolates (Table 1). The dual tropic primary isolates were also competed against specific R5 and X4 primary isolates. Finally, we performed a subset of dual infections to compete X4 versus R5 HIV-1 isolates in PBMC cultures [38], [39]. Virus mixtures were incubated with PBMC at 37°C in 5% CO_2_, washed with phosphate buffered saline (PBS) 48 hours post-infection and then resuspended in complete medium. Cell-free supernatant was collected and assayed for RT activity 5, 7, 9, 11 and 13 days post-infection. Two aliquots of supernatants and two aliquots of cells were harvested at day 13 post infection and stored at −80°C for subsequent analysis.

### Heteroduplex Tracking Assay (HTA) and Estimation of Viral Fitness

For all dual and mono-infected cultures, proviral DNA was extracted from lysed PBMC using QIAamp 96 DNA Blood Kit (Qiagen). Viral DNA was isolated from infected cells and a fragment of *env* was PCR-amplified, as described previously [39], [40]. Briefly, a set of external primers *EnvB* and *ED14*, and nested primers *E80* and *E125* were used to amplify the C2-V3 *env* region [53]. Nested PCR products of the *env* gene were analyzed by the heteroduplex tracking assay (HTA) to determine the amount of virus produced in the dual infection/competition experiments [39]. The same genomic region (*env* C2-V3) was PCR-amplified from different subtype-specific HIV-1 *env* clones [subtype A RW020 and SF170, subtype C BR025, subtype E TH22 and CAR7] [41] for use as DNA probes. However, in this case, the E80 primer was radiolabeled with T4 polynucleotide kinase and 2uCi of 5′-[γ^32^P] ATP, prior to amplification, as described [39]. At least two DNA probes in separate HTAs were used to determine relative virus production in each dual infection. Probes and PCR products were mixed, denatured and annealed on a 6% polyacrylamide gel. Heteroduplexes corresponding to each virus in a given competition were quantified using a Molecular Imager FX (Bio-Rad) imager. The final ratio of the two viruses produced in a dual infection was estimated by comparing the virus production in the competition to the virus production in the monoinfection. Production of individual HIV-1 isolates in a dual infection (*f*_0_) divided by its initial proportion in the inoculum (*i*_0_) is referred to as the relative fitness (*w = f_0_/i_0_*). Thus, the ratio of the relative fitness values of each HIV-1 variant in the competition is a measure of the fitness difference (*W_D_*) between both HIV-1 strains (*W_D_* = *W_M_/W_L_*), where *W_M_* and *W_L_* correspond to the relative fitness of the more and less fit viruses, respectively [39].

### Statistical Analysis

Statistical analyses were performed using GraphPad Prism 4 (GraphPad Software, USA). Two-tailed Student’s *t* tests were used to compare patients’ mean age. Two-tailed Mann-Whitney tests were used to compare viral load values and CD4 cell counts. Two-sided Fisher’s exact tests were used to compare winner/loser proportions in competitions between two groups of viral isolates. All tests were considered significant when p<0.05.

## Results

### Patient Clinical Characteristics

Twenty-eight HIV isolates obtained from 28 patients at various stages of HIV disease were used in this study. The mean age of subjects infected with HIV-1 subtype B and BF-like recombinants was similar (mean age 38.7 and 37.7 years, respectively, p = 0.1669, Student’s *t* test, unpaired, two-tailed) while the mean age for all 28 subjects was 38.1 (Table 1). Viral load values were also similar for both groups; subtype B and BF strains (1.2×10^5^ and 1.4×10^5^ copies/ml respectively) (Table 1). Comparison of CD4 cell counts between subtype B and BFs suggested all subjects were in late stages of disease (187 and 230 cells/μl respectively, p = 0.9399, Mann-Whitney test, two-tailed) (Table 1). Furthermore, no significant association could be established between patient viral load values (VL) and viral tropism (VL: R5 vs. X4; see below), nor between VL and subtype (data not shown).

Because drug resistance mutations affect replicative fitness of viral isolates [61], all primary isolates were screened for the presence of drug resistance mutations in the *pol* gene using the Stanford Drug Resistance Database tool (http://cpr.stanford.edu/cpr/). Two of the 28 HIV-1 isolates harbored mutations associated with high level resistance to protease inhibitors (PIs) and to reverse transcriptase inhibitors (RTIs). Namely, isolates B524 carried mutations V82A, I84V, L90M in PR, and K103N, P225H in RT while isolate BF819 had mutations D30N, N88D in PR and E101E/K and M184V in RT (Table S1).

### Phylogenetic Characterization of Argentinean HIV-1 Isolates

**Figure 2:**
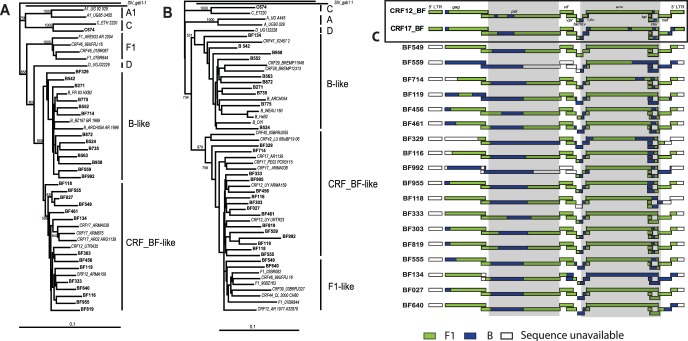
Genotypic Characterization of HIV-1 primary isolates from Argentina. Phylogenetic trees were constructed for (a) partial pol (2253–3749, HXB2) and (b) *vpu* to *env* (5968–9092, HXB2) of Argentinean primary isolates. Sequences were aligned using Clustal X and trees constructed by neighbor joining method under the Kimura 2-parameter model. (c) Sequences were aligned and bootscanned as described in materials and methods to determine recombination sites. Sequences in green represent the subtype F1 regions and in blue-subtype B. Regions in the genome that were not sequenced are represented by empty boxes. The gray shading represents those sequences used for the phylogenetic trees in 2a and b respectively.

Sequencing of two genomic fragments from a single virus facilitates subtype classification, detection of recombination sites within the HIV genome, and mapping of these recombination sites to the common breakpoints found in the BF CRF. Neighbor-joining (NJ) phylogenetic trees of partial *pol* (2253–3749, HXB2 numbering) and *vpu*-*env* (5968–9092, HXB2 numbering) sequences of these 28 Argentinean HIV-1 isolates predicted 9 (32.1%) subtype Bs, 18 (64.3%) intersubtype BF recombinants, and 1 (3.2%) subtype C (Fig. 2a, b and shaded regions of Fig. 2c; **Table 1**). Six samples (BF329, BF549, BF559, BF640, BF714, BF992) had discordant phylogenetic clustering patterns in the *pol* compared to the *vpu-env* sequences suggesting that a recombination event occurred between or within these two genes (Fig. 2a and b). Considering earlier reports of diverse BF strains circulating in Argentina [22], [42], we performed bootscan analyses on the *pol* and *vpu-env* sequences (see Materials and Methods). These algorithms confirmed the initial classification into B, F, and BF subtypes/recombinant forms as well as identified probable recombination sites, almost all of which were unique to the known BF CRFs (CRF12,17, 38, 39, 40, 42, 44, 46, and 47).

Near full length sequencing of 18 of these 28 samples confirmed results from partial *pol* and *env* sequencing, i.e. the BF recombinant patterns were different from the two main BF recombinants CRF12_BF and 17 (Fig. 2c) as well as the other BF CRFs that circulate in Argentina or other regions of South America (not shown in Fig. 2c). Only one BF recombinant, BF549, had a “pure” subtype F sequence for the *vpu* to *env* region whereas the other BF isolates had a mosaic *rev* and/or *env* composed of both subtype B and F sequences (Fig. 2c).

### Isolation of Primary Isolates, Co-receptor Usage and V3-loop Properties

Twenty-eight primary HIV-1 strains were obtained by PBMC co-cultivation using blood samples derived from HIV-1 positive patients in Buenos Aires, Argentina (Table 1). Co-receptor usage was assessed by infecting U87.CD4 cells expressing either CCR5 or CXCR4. Of the 28 primary HIV-1 isolates, 20 could only replicate in U87.CD4.CCR5 cells (R5-tropic), 4 only infected U87CD4.CXCR4 cells, and 4 were dual/mixed tropic (R5/X4) (**Table 1** **and** **2**). Additionally, only B553, B775, BF559, BF819, BF027, BF134, BF555, and BF640 replicated in MT-2 cells, i.e. the same viruses designated X4 or R5/X4 using the U87.CD4 co-receptor usage assay. The X4 and R5/X4 HIV-1 isolates were derived from late stage disease and typically had viral loads (>10^5^ copies/ml) and low CD4 cell counts (<200 cells/ml) (values not available for all patients at the time of sample collection). Two X4 isolates were subtype B and two, were BF recombinants whereas all four dual tropic viruses were BF isolates (**Table 1** **and** **2**).

The co-receptor usage of these 28 HIV-1 Argentinean isolates was confirmed by inputting the Env V3 amino acid loop sequence into various algorithms: the net amino acid charge, the 11/25 rule, Position-Specific Scoring Matrix (PSSM), and geno2pheno. It is important to note that PSSM and geno2pheno provide the highest predictive values for co-receptor usage with subtype B HIV-1 sequences as compared with other HIV-1 subtypes. The X4 and R5/X4 HIV-1 isolates had the highest positive net amino acid charge in the V3 loop when compared to that of the R5 viral isolates (mean X4 net charge = 6.75 vs. mean R5 net charge = 4.69, p = 0.0006, Student’s *t* test, unpaired, two-tailed). Three of eight CXCR4-using viruses (B775, and BF559, and BF555) had a positively charged amino acid at position 11 or 25 (306 and 322 in HXB2 Env amino acid numbering), (Table 2). In subtype B, over 90% of the X4 or dual tropic HIV-1 isolates have a positively charged amino acid at positions 11 and 25. Furthermore, the R5 isolate B563 had an Arginine at position 25, which predicts CXCR4 usage with 80–90% specificity [35]. Finally, PSSM and geno2pheno correctly predicted co-receptor usage for 26 and 28 HIV-1 isolates. Only R5 BF992 and X4 BF819 were incorrectly predicted as X4 and R5 by PSSM (respectively) whereas R5-B563 and X4-BF819 were wrongly predicted as dual and X4 tropic (respectively) by geno2pheno (**Table 2**). None of these “subtype B” algorithms were absolute in predicting co-receptor usage as determined by phenotypic assays. Limitations in predicting co-receptor usage may relate to the subtype F V3 loop sequences bore by most BF isolates (Fig. 2b and c). An antigenically distinct subtype B variant has been described in Brazil, which has a GWGR rather than GPGR signature motif at the tip of the V3-loop. This rare variant is found in ∼50% of Brazilian subtype B isolates and has been associated with slower disease progression [43]. Considering the overlap between the Argentinean and Brazilian HIV epidemics, we analyzed the diversity of the V3-loop. Only one of the Argentinean HIV sequence (BF134) had a GWGR motif, which was also the only BF strain with a subtype B Env gp120 coding region. The subtype F V3 sequences of the BF strains had either a GPGR or GPGQ sequence, the latter being more common for subtype F but infrequent for subtype B [44].

The N-linked glycosylation site, N301, is part of a highly conserved NNTR sequence found in the V3 loop of CCR5 tropic primary isolates [45]. All of the subtype B, F, and BF sequences had NNTR sequence with the exception of two X4 isolates, which harbored either NTIKQ (B552, with an isoleucine insertion) and NKTQ (B775) (**Table 2**). Aside from N-linked sites at amino acid position 301, no significant differences were observed in the sequence lengths of any variable loop or in the number of N-linked glycosylation patterns in the V1–V5 regions with the Argentinean subtype B and BF isolates.

### Relative Replicative Fitness of Primary Subtype B, C, F and Intersubtype BF Recombinant HIV-1 Isolates of Different Phenotypes

**Figure 3:**
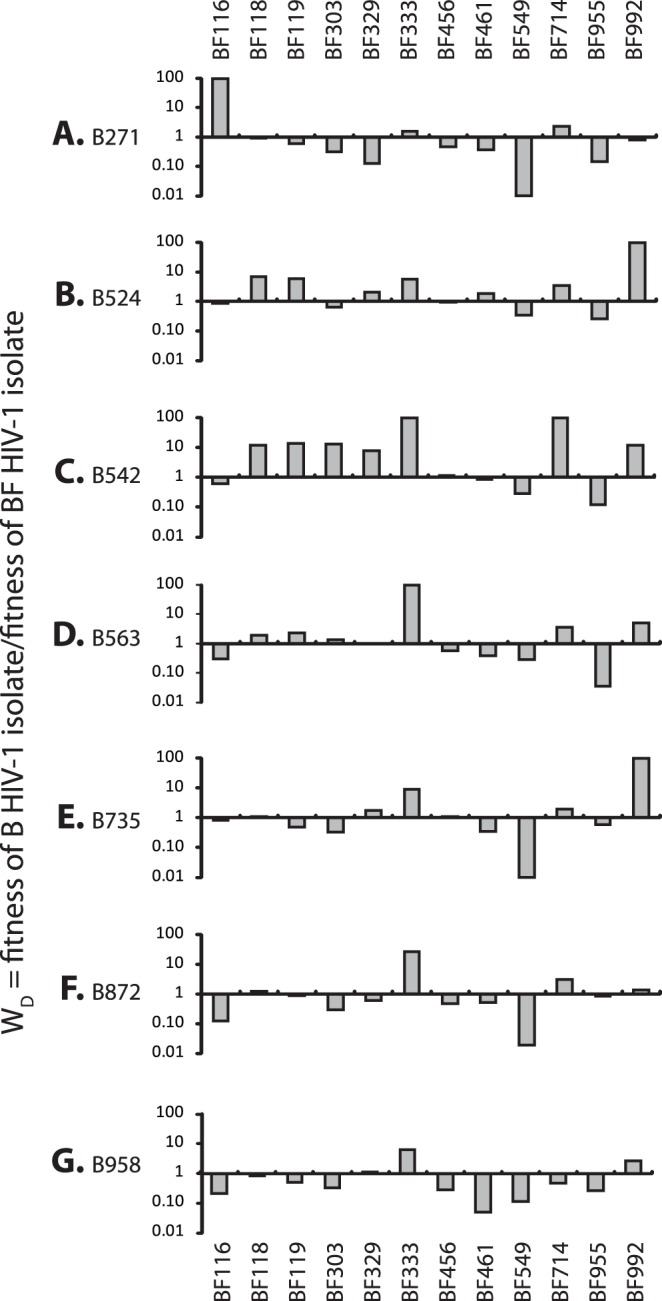
Comparing the relative replicative fitness difference (W_D_) of HIV-1 subtype B and BF recombinants from Argentina in direct competitions. Subtype B strains a) B271 b) B524, c) B542 d) B563 e) B735 f) B872 g) B958 were competed against CRF-BF-like isolates (BF116, BF118, BF119, BF303, BF329, BF333, BF456, BF461, BF549, BF714, BF955, BF992) in PHA-stimulated, IL2-treated PBMCs. The fitness difference represented on the Y axis ranges from 0.01 to 100. Bars represent the fitness of the isolates shown on the Y-axis.

**Figure 4:**
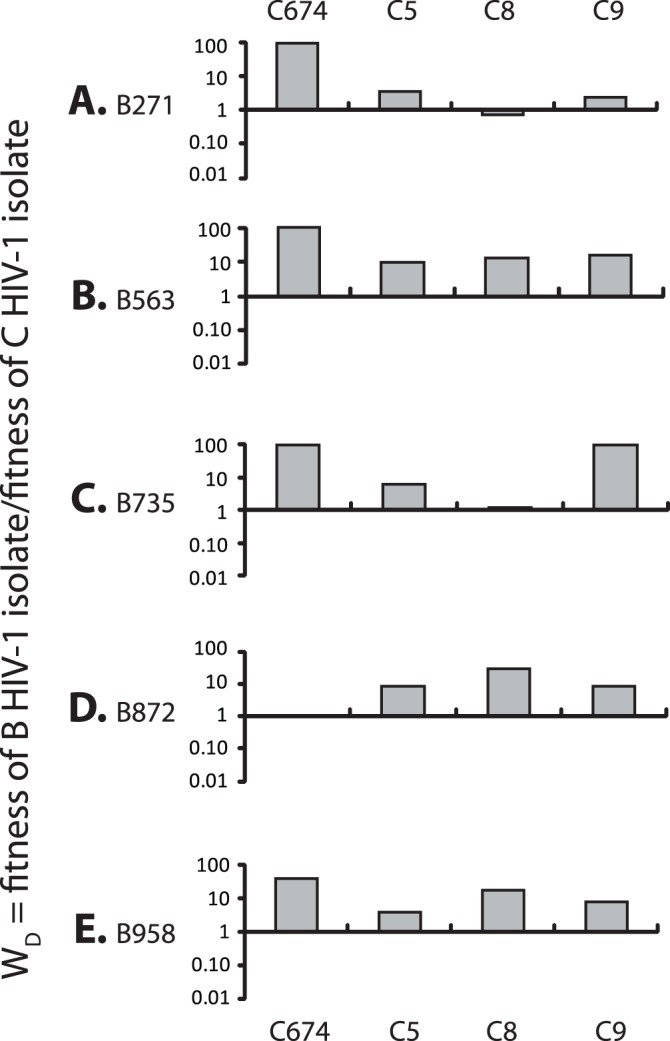
Relative fitness difference (W_D_) between Argentinean subtype B strains a) B271, b) B563 c) B735 d) B872, e) B958 and Subtype C strains C674 (Argentina), C5, C8, C9 (reference strains). Competitions were performed in PHA/IL2 treated PBMCs as in Figure 3. The fitness difference shown on the Y axis ranges from 0.01 to 100. Bars represent the fitness of the isolates shown on the Y-axis.

**Figure 5:**
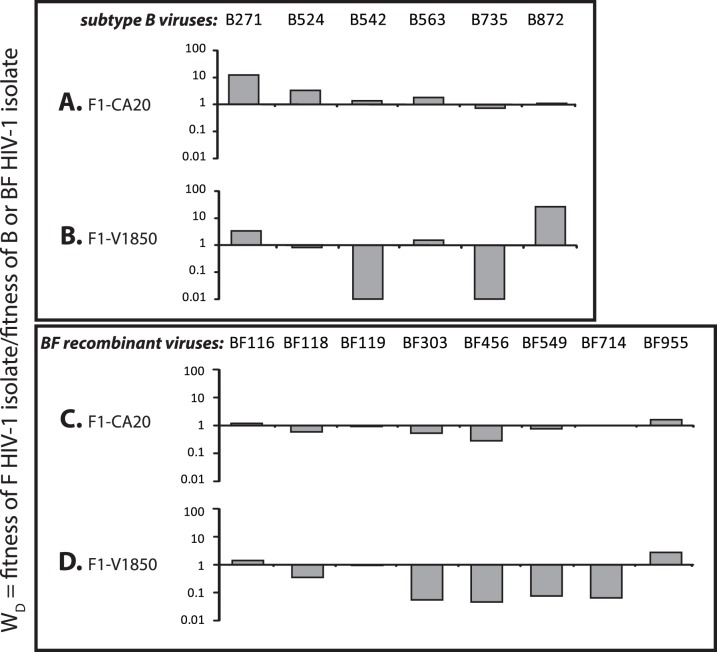
Comparing the relative replicative fitness difference (W_D_) of HIV-1 sub-subtype F1 reference strains; (a) CA20 and (b) V1850 and Argentinean subtype B (B271, B524, B542, B563, B735 and B872) in direct competitions using PHA/IL2 PBMCs. Similarly, subtype F (c) CA20 and (d) V1850 were competed against BFs (BF116, BF118, BF119, BF303, BF456, BF549, BF714 and BF955). Bars represent the relative fitness of the viruses shown on the Y-axis.

**Figure 6:**
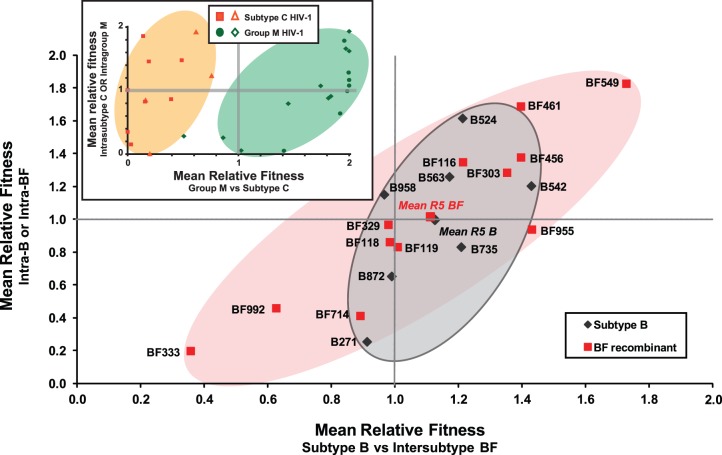
Comparison of intra- and intersubtype fitness of R5 HIV-1 subtype B and various BF recombinants from Argentina. Mean relative fitness values were determined for each HIV-1 isolate from pairwise competitions between isolates of the same subtype (mean intrasubtype relative fitness) and of different subtypes (mean intersubtype relative fitness). The mean intrasubtype and intersubtype fitness values for each HIV-1 isolate were then plotted as the *x* and *y* coordinates. Subtype B viruses are shown as black filled squares while BF recombinants are indicated by a red filled square. The mean fitness of subtype B and BF are very similar and also indicated as red and black filled squares respectively. The insert figure represents the fitness of HIV-1 subtype C relative to group M strains as described in a previous study (Abraha et al, 2009). Subtype C strains (red squares and triangles) have low mean relative fitness and cluster together (orange oval shade) while group M strains (green dots and tetragon) are more fit and cluster together as well (green oval shade).

**Figure 7:**
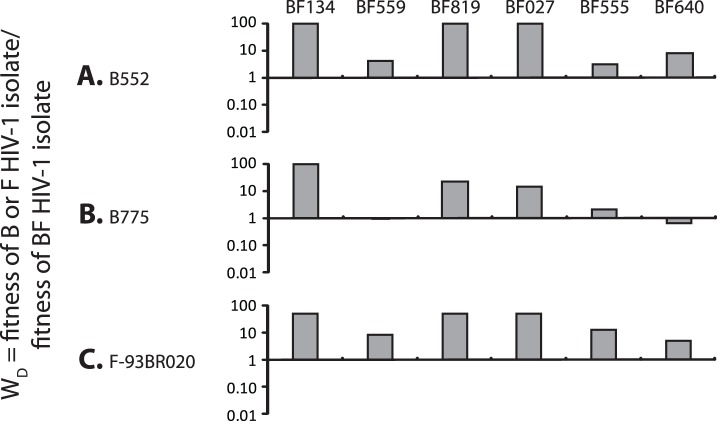
Comparing the relative replicative fitness difference (W_D_) of syncytium inducing (SI) HIV-1 Argentinean subtype B (a) B552, (b) B775 and (c) reference subtype F, 93BR020 against BF Argentinean recombinants (BF134; BF559; BF819, BF027, BF555 and BF640) in direct competitions using PHA/IL2 PBMCs. Bars represent the fitness of the viruses shown on the Y-axis.

In order to determine the *ex vivo* pathogenic fitness of primary R5 and X4 subtype B, C and intersubtype BF recombinant isolates from Argentina, nearly 700 dual infection/competitions experiments were performed in PHA stimulated human PBMCs using the 28 primary HIV-1 isolates (described above) and 6 reference strains of subtypes C (n = 3); F1 (n = 2), and F2 (n = 1) (**Table 1**). Full pairwise competition experiments were performed with the 25 primary R5 isolates and the 9 primary X4- and X4/R5-tropic isolates (Figures 3–7). The fitness difference, W_D_ is defined as the fitness of a B isolate over the fitness of BF isolate or C isolate in PBMC competitions (Fig. 3 and 4, respectively). In Fig. 3, we show that the replicative fitness of each R5 subtype B isolate (y axis of panels A–G) is, on average, similar to each of the 12 BF isolates in these pairwise competitions. Only one BF isolate (BF549) is consistently more fit that all the B isolates (Fig. 3a–g) and other BF strains (Fig. S1a). Intersubtype BF recombinants and subtype B isolates had nearly equal number of “wins” in direct head-to-head competitions (BF wins: 55.95%, n = 47 while B wins: 44.05%, n = 37, p = 0.1647). However, as shown in Fig. 4, the subtype B and BF isolates clearly out-compete the subtype C HIV-1 isolates with C viruses winning only one of 22 head-to-head competitions (p<0.0001, two-sided Fisher’s exact test) confirming previous observations that subtype C strains are less fit than most HIV-1 group M strains [36], [40], [46]. The only Argentinean subtype C isolate in our panel, C674 was the least fit compared to the other 3 C HIV-1 isolates derived from South Africa, Nigeria and Malawi (Fig. 4).

HIV-1 subtype F1 is a parental subtype of BF recombinants. However, “pure” subtype F HIV-1 is now rare in Argentina as reflected by its absence in our set of Argentinean primary isolates and recent molecular epidemiology studies [16], [42] (Fig. 1). We therefore included a CCR5-using subtype F1 primary isolate, CA20 from Cameroon and subtype F2, VI850 from the Democratic Republic of Congo (DRC) for competitions against 6 Argentinean subtype B (Fig. 5a and b) and 8 BF strains (Fig. 5c and d). The sub-subtype F1 is most related to South American F isolates and the F segments in BF recombinant genomes. The F1 isolate, CA20 was of equal or slightly greater fitness than the subtype B and BF isolates whereas the F2 isolate, VI850 was slightly less fit and lost the majority of competitions against the subtype B and BF isolates from Argentina. Nonetheless, there was no significant difference in replicative fitness between any of these subtype B, F, or BF HIV-1 isolates.

The mean intra- and intersubtype relative values were plotted for each of the B and BF isolates (Fig. 6) to obtain a clear representation of relative replicative fitness in human PBMCs. In Fig. 6, the mean intra-subtype/-recombinant relative fitness values (y axis) represents the average fitness of each B (or BF) isolate against each of the other B (or BF) isolates. Inter-subtype/-recombinant (x-axis) represents the average fitness of each B (or BF) isolate against each of the BF (or B) isolate. When comparing the intra- and inter-group M and subtype C fitness of 29 primary HIV-1 isolates in a previous study, we clearly showed that the subtype C isolates were on average less fit than any group M isolate (e.g. subtype A, B, D, and CRF01_AE) (see insert panel A of Fig. 6). In contrast, the replicative fitness of the subtype B and BF isolates from the inter-subtype/-recombinant comparisons overlapped with the intra-subtype fitness comparisons. In other words, some isolates such as BF549 had both high intra- and inter-subtype/-recombinant fitness but on average the BF isolates were not more fit than B isolates or vice versa (see mean R5 B fitness vs mean R5 BF fitness in Fig. 6).

A more limited set of CXCR4-using subtype B (B552, B775) and BF (BF134, BF027, BF555, BF640) HIV-1 isolates were available from Argentinean patients for these fitness studies. These viruses as well as one subtype F X4 reference strain (93BR020) were used for pairwise competitions in PBMCs (Fig. 7, Fig. S1b). As described in Fig. 7, it appears that X4 B isolates had higher fitness than the BF HIV-1 isolates in direct head-to-head competitions (B wins 10 of 12 competitions, 83%, p = 0.0033). However, the BF819 isolate is an X4 isolate carrying drug resistance mutations D30N and N88G in PR and M184V in RT. The lamivudine-resistant M184V mutations is associated with a fitness cost [47]. When excluding the BF819 isolate from these analyses, there was no statistical difference in replicative fitness between these CXCR4-using subtype B and BF isolates. The sole subtype F X4 isolate was more fit than all the BF recombinant and B isolates (not shown). In summary, a total of 40 competitions between the other two subtype F isolates, 9 subtype B isolates, and 18 BF recombinants show similar replicative fitness (p = 0.129) and suggest that this X4 subtype F isolate, 93BR020 and the R5 BF549 isolates were outliers with high replicative fitness.

Regardless of subtype, X4 HIV-1 isolates usually replicate faster and to higher levels than R5 HIV-1 isolates in PBMC cultures [39], [46]. However, as previously reported [36], [39], [46]), these dual-infection experiments in PBMC cultures do not represent “true” competitions as R5 and X4 isolates do not compete for the same susceptible cells. CCR5 and CXCR4 are typically expressed on different CD4+ T-cells (predominantly on memory and naïve, respectively) [48] or at different levels in macrophages (higher CCR5 over CXCR4 levels). In this study, two R5 subtype B and one R5 intersubtype BF isolate were competed against three X4 primary isolates (1 subtype B and 2 intersubtype BFs isolates). All X4 isolates out-produced R5 variants, in a subtype independent manner, further supporting the higher pathogenic fitness of X4 isolates over the R5 HIV-1 isolates in PHA-activated, IL2-maintained PBMC cultures (Data not shown).

## Discussion

In many epidemics around the world, generation and transmission of intersubtype HIV-1 recombinants appears to outpace the expansion of the “pure” parental subtypes. For example, BC recombinants are now increasing in prevalence as compared to subtype B or C HIV-1 isolates in Rio Grande do Sol province in Brazil [49]–[51] (Fig. 1). BC recombinant forms (specifically CRF07 and 08) dominate over subtype B’ or C in China [52]. Finally, the HIV-1 epidemic in Argentina involves a complex mixture of BF recombinants expanding faster than subtype B infections [42] (Fig. 1). This increasing prevalence of recombinant forms might infer greater replicative fitness over the parental HIV-1 subtype in the epidemic. However, “fitness” in the HIV-1 epidemic is not necessarily comparable to *ex vivo* replicative fitness in PBMCs whereas virulence (i.e. rate of disease progression within a patient) is a direct correlate of replicative fitness [53]. With the exception of studies comparing the relative fitness of subtype A, G, and CRF02_AG HIV-1 isolates [54], [55] *ex vivo* replicative fitness of intersubtype HIV-1 recombinants have not been compared to their parental HIV-1 subtypes in primary human cells.

To obtain a preliminary understanding on how HIV-1 recombination in the epidemic impacts replicative fitness (and possibly pathogenesis), we compared the *ex vivo* fitness of reference subtype F, primary isolates of subtype B, and recombinant BF HIV-1 from Argentina where a significant emergence of BF recombinants is recent (mid 1990s–early 2000s) and complex [42], [56], (Fig. 1). Initial V3 loop genotypic analyses of HIV-1 in Argentina suggested that the epidemic was similar to that in North America where subtype B was and still is highly prevalent [57]. However, we now know that subtype F HIV-1 may have a longer history in Argentina, having been introduced between 1975–1980 [56]. Recent studies have shown that the HIV epidemic in Argentina is dominated by pure subtype B, various BF recombinant forms, and a rare occurrence of subtypes C and F. Accordingly, we were unable to identify a “pure” subtype F isolate among 28 HIV infected individuals which is consistent with recent reports of rare subtype F HIV-1 infections in Argentina [42], [58]. Early in the Argentinean epidemic, subtype B was most common in men who have sex with men (MSM) and subtype BF among IDUs and the heterosexually-active population [59], [60] but differential prevalence of subtypes/recombinants in these transmission groups has eroded over the past decade. Despite attempts to classify the BFs as CRFs, our analyses of 18 full and near full length BF genomes revealed that most BF breakpoints did not match the recombination sites used to classify CRF12,17, 38, 39, 40, 42, 44, 46, and 47. In the past, a majority of BF recombinants isolated from HIV-infected Argentineans have been classified as CRF12_BF and CRF17_BF based on partial pol sequences [17], [22], [42]. We are now analyzing all of the full length BF sequences in the Los Alamos HIV-1 sequence database (n = >40) and preliminary results suggest that the vast majority of the BF strains classified as a CRF might instead be URFs of recent B+F or BF+B co-infections, the latter being the most likely. We observed little evidence of an epidemic with stable BF CRFs in Buenos Aires. These findings are consistent with recent studies showing a decrease in CRF12_BF prevalence (69% to 46% from 1986–93 to 2001–2008) [42] and an increased prevalence of unique BF recombinant forms in Argentina (up to 50% infections by URF_BF) [17], [42] (summarized in Fig. 1). The later introduction of subtype C in Argentina from Brazil has led to the identification of BC recombinants (which may have originated in Brazil) and of a complex B/C/F recombinant in one patient [61].

Does the predominance of BF recombinants suggest a better fitness of these strains? Factors such as founder effects, host restrictive factors as well as cultural/behavioral factors may affect differential global spread of HIV variants in the human population [3], [4], [62]. We are also adopting infectious disease models to understand HIV spread in the epidemic [63]. Lower virulence (related to reduced replicative fitness in PBMCs [64]) leads to longer asymptomatic infection and increased opportunity for transmission. Aside from an initial founder event(s), the other key factor in these models is transmission efficiency related to the amount of transferred virus (e.g. viral load in donor [65], [66]) and the ability to establish new host infections (e.g. relative replicative capacity and transmission through mucosal/genital tissue [36], [38], [40]. The dominance of BF-like recombinants over subtypes B, C and F in Argentina may be related to some biological advantage such as better transmission efficiency coupled with weaker virulence over the parental “pure” subtypes B and F. A second but not exclusive factor relates to the timing of founder events into the Argentinean population, and possibly into different transmission groups [49]. As described above, with the continuous emergence of new BF recombinants, these different transmission groups (e.g. IV drug users, heterosexuals, and MSMs) have now mixed and the BF epidemic in Argentina is now driven by heterosexual transmissions. This study only suggests that the similar replicative fitness of B, F, or BF HIV-1 isolates may relate to similar rates of disease progression (as determined by increasing viral loads and decreasing CD4 cell counts). Thus, we propose that the increased spread of BF isolates in the Argentinean epidemic may be related to better transmission opportunity and/or efficiency of BF over B or F HIV-1 isolates. We are currently testing transmission efficiency in our ex vivo transmission models involving infections of human penile, cervical, vaginal, and rectal explants.

After >700 head-to-head competitions between 9 B, 18 BF, 3 F, and 4 C HIV-1 isolates (28 from Argentina), it was clear that subtype B and BF HIV-1 isolates had similar replicative fitness in human PBMC cultures. Subtype C HIV-1 isolates were less fit than all of the 27 B, BF, and F HIV-1 isolates, which remained consistent with multiple studies showing poor replicative fitness of subtype C HIV-1 compared to any other group M HIV-1 isolate (derived from >2000 pairwise competitions employing over 50 primary HIV-1 isolates) [36], [40], [46]. Our studies and those of others have suggested that HIV-1 subtype F isolates may be of slightly higher replicative fitness than the majority of group M isolates. The proposed higher replicative capacity of F and BF HIV-1 strains is based on specific gene sequence or other genetic factors. One study suggested that the subtype F long terminal repeat (LTR) showed greater transcriptional activation than the LTRs of subtype B [25], possibly leading to higher levels of virus production. Aulicino et al (2007) [58] examined 40 Argentinean *vpu* sequences of BF strains and revealed a high substitution rate (∼11×10^−3^ substitution per site per year) than that observed among subtype B strains [67]. These studies were limited to one or few B, BF, or F sequences and did not examine the effects of these gene regions/sequences in the context of the entire virus isolate. Nonetheless, both studies provide some evidence for increased subtype F fitness over subtype B HIV-1 isolates.

Based on the cumulative data from over 5000 pairwise competitions in PBMCs [36], [40], [46], we now rank replicative fitness of subtypes and dominant CRFs as follows: B = D = F>CFR02_AG≥A = CRF01_AE>>C. In addition there is a strong correlation between relative replicative fitness in PBMCs and virulence in humans. Interestingly, the subtypes of highest replicative fitness (B, D, and F) are also the viruses expanding the slowest in numerous regional pandemics resulting in apparent shifts in the dominant subtypes, e.g. CRF01_AE or URFs over subtype B in Thailand, subtype C-containing URF and subtype C over subtype B in Brazil, and BF URFs over subtype B and F in Argentina. We suspect that these subtype/CRFs/URFs of lower virulence are expanding due to greater opportunity for transmission and retention of high transmission efficiency. Preliminary studies suggest that all HIV-1 subtypes in group M have similar transmission fitness when employing human genital tissue as a model for primary infection. As with the near extinction of HIV-1 subtype F in the Argentinean HIV-1 epidemic over the past 40 years, the dominance of subtype F HIV-1 over other subtypes has also decreased in Romania [68] and is almost absent in Cameroon (5% in 1999); [27] despite the moderate frequency of subtype F1 (17% of all HIV infections) during the late 80’s and early 90’s [69]. Increase in prevalence and new infections by a specific subtype may simply be due to founder events in a specific transmission group. Nonetheless, we consistently observe that HIV-1 subtypes (D, F, G, and CRF01_AE) that have decreased in prevalence (in Uganda, Romania/Argentina, Cameroon, and Thailand, respectively) also have the highest ex vivo replicative fitness.

## Conclusion

The present study suggests that BF recombinants circulating in Buenos Aires, Argentina have multiple recombinant structures which most likely resulted from continuous dual/superinfection between B and BF. Further, similar replicative fitness between subtypes B and BF HIV-1 isolates implies that recombination of the parental HIV-1 isolates does not necessarily increase replicative fitness. Initial dual infection or superinfection may result in higher fitness based on immune escape, host adaptation, and/or increase replicative fitness. Subsequent transmission of the recombinant form (e.g. BF) may select for factors associated with high transmission efficiency, which appear to be different than those factors governing replicative fitness in PBMCs. It is worth noting that CRF02_AG, another dominant recombinant was found to be more fit than its parental subtypes A and G, [54], [55] suggesting that this higher replicative fitness may be associated with its dominance in the African HIV epidemic. However, the origin of CRF02_AG (or sub-subtype A3) dates back to the start of the HIV-1 pandemic in the Congo basin whereas our BF strains may have originated from very recent dual or super infections in Argentina [15]. Following approximately 50 years of evolution, the replicative fitness of CRF02_AG may reflect the changes derived from the original recombination as well as the accumulation of stable substitutions within the genome. With the analyses of the BF recombinants, evolution may be limited to just one or few transmission events.

## Supporting Information

Figure S1 **Fitness difference for pairwise competition with R5 and X4 HIV-1 isolates.** Fitness differences (WD) for pairwise competition with A) R5 and B) X4 HIV-1 isolates are shown as the relative fitness of the “column” isolate over the “row” isolate. Gray boxes represent intra-subtype fitness differences while unshaded boxes represent inter-subtype fitness differences among HIV-1 patient’s isolates and reference strains from subtype B (bold italic), intersubtype BF recombinants (bold), subtype C (italic) and subtype F (normal).
(XLSX)

Table S1 **Drug resistance mutations: calibrated population resistance tool from Stanford University was used to evaluate the presence of drug resistance mutations in** ***pol*** **gene sequence from each viral isolate (2253–3749, HXB2 numbering).**
(XLSX)
